# Design of reinforcement learning for perimeter control using network transmission model based macroscopic traffic simulation

**DOI:** 10.1371/journal.pone.0236655

**Published:** 2020-07-30

**Authors:** Jinwon Yoon, Sunghoon Kim, Young-Ji Byon, Hwasoo Yeo

**Affiliations:** 1 Department of Civil and Environmental Engineering, Korea Advanced Institute of Science and Technology, Daejeon, Republic of Korea; 2 Department of Civil Infrastructure and Environmental Engineering, Khalifa University of Science and Technology, Abu Dhabi, UAE; Central South University, CHINA

## Abstract

Perimeter control is an emerging alternative for traffic signal control, which regulates the traffic flows on the periphery of a road network. Some model-based approaches have been suggested earlier for the optimization of perimeter control based on macroscopic fundamental diagrams (MFDs). However, there are several limitations when considering their application to a large-scale urban area because the model-based approaches may not be scalable to multiple regions and inappropriate for handling various effects caused by the shape change of MFDs. Therefore, we propose a model-free and data-driven approach that combines reinforcement learning (RL) with the macroscopic traffic simulation based on the recently developed network transmission model. First, we design four perimeter control models with different macroscopic traffic variables and parametrizations. Then, we validate the proposed models by evaluating their performances with the test demand scenarios at different levels. The validation results show that the model containing travel demand information adapts to a new demand scenario better than the model containing only density-related factors.

## Introduction

Perimeter control is proposed as an alternative approach for traffic signal control, as adaptive traffic signal control (ATSC) [1,2] has a critical limitation that it is less effective when the traffic demand is oversaturated [3]. Perimeter control is a type of traffic demand control that regulates the regional traffic flows on the periphery of a road network to maintain a proper level of service. Its key concept is suggested by Daganzo in 2007 [4] and it is established using macroscopic fundamental diagrams (MFDs) [5,6]. As MFDs give a macroscopic relationship between the regional vehicle accumulation and the network outflow in an aggregated manner, the potential action for perimeter control can be determined on its basis.

Several studies focus on the optimization of perimeter control by recognizing the significant relationship between the macroscopic variables of MFDs. As found in the literature, the model-based approaches are conventionally used, typically the feedback control and the model predictive control (MPC). Feedback control determines the optimal level of boundary capacities based on MFDs according to the current state accommodating the vehicle accumulation to the critical density that maximizes the network outflow. On the other hand, MPC provides a multi-stage optimal solution to the sequence of states predicted based on the state transition model.

The conventional model-based approaches for the optimization of perimeter control have been effectively applied to some relatively small-sized areas, such as the gating control of a single protected network (PN) [7–9] or one-to-one transfer flow control for two regions [10,11]. However, there has been a growing interest in applying perimeter control to a widened urban area in recent studies [12–16]. Accordingly, some challenges regarding the conventional model-based approaches are newly found when considering the application of perimeter control to a large-scale urban area.

First of all, both feedback control and MPC may not be scalable, which makes them ineffective when optimizing multi-regional perimeter controls. For instance, although feedback control has relatively cheap computational costs, it does not provide a multi-stage optimal solution to the multiple agents since it only maximizes the instantaneous network throughput based on MFDs. MPC can provide multi-stage optimal solutions, on the other hand, the computational cost would dramatically increase as the number of control agents increases [17]. Second, the conventional approaches may be inappropriate for handling the effects caused by the shape changes of MFDs. In most cases, the conventional approaches assume that the shape of MFD is well-defined and steady-state. However, several studies have proven that there are a number of factors that affect the shape changes of MFDs [18–26]. There have been some attempts to handle the effects of shape changes using closed loop structure of MPC [27], since the efficiency for the control action is dependent on the precise estimation of MFDs. However, the dynamics would become more complex as the spatial range of the control area expands, resulting in an exponential increase of the computational burden.

Therefore, we propose a model-free and data-driven approach using reinforcement learning (RL) as an effective alternative for perimeter controls on a large-scale urban area. RL is an emerging technique applied in the field of traffic signal control with the advantages explained as follows: it is known that the well-trained RL models can perform the multi-stage and high-level optimization at par with MPC with lower computational costs [17]. Thus, RL would be a scalable method for the multi-regional perimeter control. Moreover, RL can better cope with the effects caused by the shape changes of MFDs in conjunction with data-driven methods. Additionally, the online learning framework of RL improves the control efficiency when it is applied in real-time.

Despite several advantages of RL, few relevant studies can be found in the literature because there had been no proper macroscopic traffic simulation to train the perimeter control. A traffic simulation is essential for training the signal control model, however the conventional simulators have a limitation in delicately describing the macroscopic behaviors of the transfer flows between multiple networks. A few studies focus on the signal control of several number of urban intersections in a single traffic network, but they do not consider the network flow control between multiple networks [28–30]. Fortunately, some recent studies, such as Kim *et al*. [18,19] and Knoop & Hoogendoorn [31], provide an elaborate analysis of macroscopic behaviors of transfer flow based on MFDs, which enables us to develop an improved macroscopic traffic simulation. Thus, this motivates the development of RL-based perimeter controls for a large-scale urban network.

Hence, this research aims to design an effective RL model for perimeter control using a newly developed macroscopic traffic simulation based on the network transmission model. The scope for the model design includes the selection of variables and the method for the parametrizations. We experimentally validate the proposed models under a well-controlled environment, since there is no relevant study for constructing an RL-based perimeter control. In this article, the literature review for perimeter control is provided. Then, we construct four different models for perimeter control using different model designs in order to analyze the effectiveness of each parameter. Finally, the proposed models are experimentally validated with different types of test demand scenarios to investigate the models’ ability to adapt to a new situation.

## Literature review

Traffic signal control is considered as an effective method for resolving urban congestion problems and it has been continuously studied since the 1950s. Adaptive traffic signal control (ATSC), developed in the 1990s, is the latest system that is still in use for many cities internationally [1,2]. However, there remains a known major drawback of ATSC, that it is less effective when the traffic demand becomes oversaturated occurring spillback or gridlock [3]. To overcome this limitation, perimeter control approach is newly proposed in recent years and newly developed MFDs became a theoretical basis for perimeter control.

A well-defined MFD gives a unimodal and low-scattered relationship between the network outflow and vehicle accumulation in an aggregated manner that enables to measure the traffic state of a relatively wide area. Since the control action for perimeter control is highly dependent on MFDs, a number of researches have been conducted to identify the causal factors influencing the shape changes of MFDs. Several studies [20–22] point out that the shape of MFDs can be scattered by the spatial inhomogeneity of the traffic densities and its effects have been analyzed in [21,23,24]. In addition, a few studies [25,26] focus on the hysteresis phenomena of MFDs during the network-wide traffic loading and unloading phases. Moreover, the effect of varying hourly traffic demand patterns and the change of boundary capacity due to the control policies have recently been examined as additional factors of shape changes [18,19].

Several subsequent studies focus on the application of perimeter control on the traffic networks by recognizing the significant relationship of MFDs. In the early years, gating control emerges as a simple type of perimeter control. It aims to regulate the regional inflow properly towards a small unit of traffic network, so-called protected network (PN), to prevent the central business district (CBD) from being oversaturated [7–9,32]. However, gating control has a limitation that it does not consider the control effect on the adjacent outer network. As there is a growing interest in developing a multi-regional boundary control, recent studies focus on the transfer flow control between the boundaries of the multiple networks by regulating both inflows and outflows [12–15,33,34]. Perimeter control is a generic concept that includes both gating control and multi-regional transfer flow control.

In order to optimize the control action, feedback control and MPC are typically used in most of the literature. Feedback control determines the optimal level of boundary capacities based on MFDs according to the current state accommodating the vehicle accumulation to the critical density that maximizes the network outflow. Several methods, such as a proportional-integer (PI) [7,35,36], linear-quadratic-integer (LQI) [33], or linear-parameter-varying (LPV) [9] are used for feedback control. On the other hand, MPC based control focuses more on the multi-stage optimization itself by formulating the objective functions in terms of the performance measures such as maximization of outflow [27], minimization of total time spent [14], total delay [15], or total travel time [37].

Although these model-based approaches have been effectively applied to some relatively small-sized areas, it is found that the spatial range for perimeter control expands in recent studies. One can notice that the early studies focus on single gating controls as in [7–9] or a small number of networks as in [10,11], whereas recent studies expand their spatial range to multiple boundary control problems as in [12–16], using mostly feedback control as the computation cost is cheaper than that of MPC. However, it requires to develop a scalable and effective model for a large-scale urban area, since feedback control may not provide a multi-stage optimal solution for the multiple agents.

## Construction of perimeter control models

In this section, four models using different designs are proposed for perimeter control. The scope for the model design includes the selection of variables and the method of parametrizations. The first one is a baseline model that defines its state variable with only density-related factors, similar to the prevalent methods found in the literature on the RL-based traffic signal control of individual traffic lights [28,29,38,39]. The other three models contain the travel demand information as the explanatory variables.

### 1. Construction of the baseline model

The design of the baseline model follows the prevalent methods found in the literature on the RL-based traffic signal control of individual traffic lights. First, the state variable is defined with a pair of vehicle accumulations of the adjacent networks, which is a representative macro-scale density-related factor. Next, the action is defined by the green split ratio as in (1),

$$a(t)=\frac{gt(t)}{c}$$ (1)

where *c* is the cycle time and *gt*(*t*) is the green signal time for the direction of the transfer flow. This means that we consider ‘green time ratio’ with the fixed cycle time as the control variable in this study. Then, the chosen action value determines the boundary capacity for the transfer flow as in (2),

$$C_{i\to j}(t)=\sum_{h\in H}{f_{s}^{h}}_{i\to j}∙a(t)∙l_{h}$$ (2)

where *C*_*i*−*j*_(*t*) represents the boundary capacity between network *i* and network *j* at time *t*, ${f_{s}^{h}}_{i\to j}$ is the saturation flow rate, *H* is the set of links that connect the network *i* and *j*, and *l*_*h*_ is the number of lanes of the link *h*∈*H*. The details can be found in [18].

**Fig 1:**
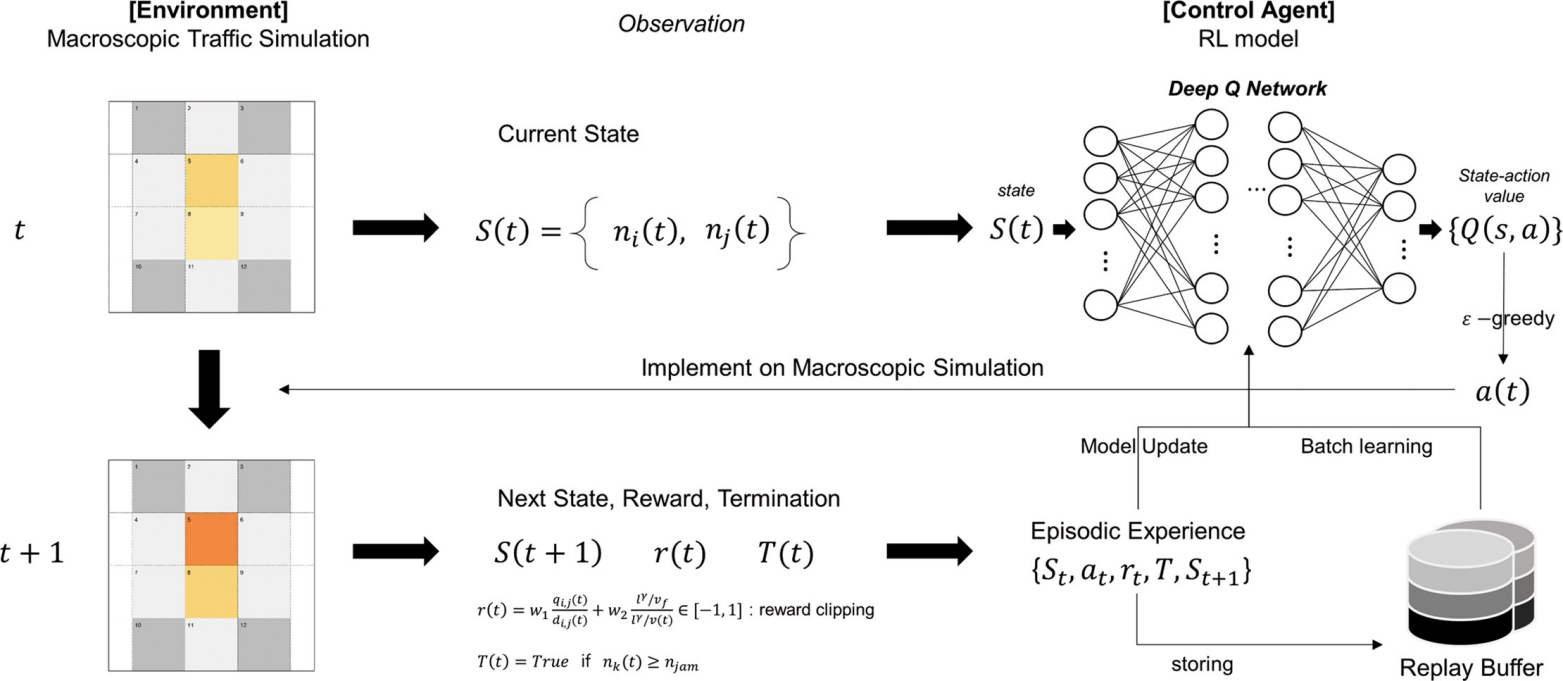
Training framework of the RL-based perimeter control model. (Drawn by authors).

The reward is defined by a linear combination of the network throughput and the total delay time which measures the local- and system-level of performances, respectively. Then, the reward value is rescaled to [−1,+1], as in [40]. Moreover, the termination is also used as an identifier of the oversaturation to prevent the target networks from being in a gridlock or spillback. If one of the networks falls into a gridlock during the training, then the corresponding episode is ended by this termination giving the penalty of −1 to the reward. Fig 1 shows the training framework for RL-based perimeter control using the macroscopic traffic simulation.

### 2. Parametrization of demand information as explanatory variables

Since there has been no relevant reference of the effective designs for perimeter control model in the literature, we propose three different designs by considering the information of the travel demand pattern as the explanatory variables. These models append the parameters for the travel demand information to the state of the baseline model which only contains the density-related factors. According to the type of variables and the method of parametrizations, the proposed models are classified by referring as PD-, FD-, and MD-models. PD-model contains the partial information of the transfer flow between two adjacent networks, while FD- and MD-model contain the full demand information in their state. In addition, MD-model modifies the parametrization method of the FD-model in order to exclude a newly generated quantity of traffic demands.

#### Model 1: PD-model

The first model appends the partial information of the transfer flow to the state variable of the baseline model. The underlying hypothesis is that the transferring demand between the two networks represents the travel demand pattern of the whole network. Therefore, the state variable is defined by a four-dimensional vector as in Eq (3),

$$s(t)=[n_{i}(t),n_{j}(t),f_{i,j}(t),g_{j}(t)]$$ (3)

where *n*_*i*_(*t*), *n*_*j*_(*t*) are the vehicle accumulations, *f*_*i*,*j*_(*t*) is the transfer demand of the source network, and *g*_*j*_(*t*) is the supplying ability of the sink network. Let this model be referred as a PD-model, which stands for a **P**artial **D**emand model.

#### Model 2: FD-model

Let the second model be an FD-model, which stands for a **F**ully-supported **D**emand information model. This model contains every directional travel demand of in/out flow of two adjacent networks as in (4),

$$s(t)=\left[n_{i}(t),\{d_{i,in}^{N_{i}}(t)\},\{d_{i,out}^{N_{i}}(t)\},n_{j}(t),\{d_{j,in}^{N_{i}}(t)\},\{d_{j,out}^{N_{i}}(t)\}\right]$$ (4)

where $\left\{d_{∙,in}^{N_{i}}(t)\},\{d_{∙,out}^{N_{i}}(t)\right\}$ represent a set of the directional travel demand for the in/out-flow of the given network toward $N_{i}$, and $N_{i}$ is a set of neighboring networks of *i* containing *i* itself. Note that $d_{i,in/out}^{i}(t)$ means the local in/out-flow of the network *i*. The underlying hypothesis is that the partial information of demand patterns does not represent the demand pattern of the whole network, which is contrary to that of the PD-model.

#### Model 3: MD-model

Let the third model be a MD-model, which stands for a **M**odified F**D**-model. This model uses the same structure with the FD-model, however this model modifies the demand value at the next time step $d_{∙∙}^{N_{i}}(t+1)$ to consider the net-quantity excluding a newly produced travel demand. The underlying hypothesis is that the travel demand factors in the state variable should not contain any exogenous disturbances, such as a newly generated in/out-flow at the next time step. Thus, the re-formulation is used as in (5),

$${\tilde{d}}_{∙∙}^{N_{i}}(t+1)≔d_{∙∙}^{N_{i}}(t)-T(d_{∙∙}^{N_{i}}(t))$$ (5)

where $T(d_{∙∙}^{dir}(t))$ represents the quantity of the transfer flow determined by the chosen action value using the network transmission model [18]. Then, with this notation, the state variable of MD-model is expressed as in (6).

$$\tilde{s}(t+1)=\left[n_{i}(t),\{{\tilde{d}}_{i,in}^{N_{i}}(t)\},\{{\tilde{d}}_{i,out}^{N_{i}}(t)\},n_{j}(t),\{{\tilde{d}}_{j,in}^{N_{i}}(t)\},\{{\tilde{d}}_{j,out}^{N_{i}}(t)\}\right]$$ (6)

In this case, the reformulated state variable in (6) may result in $\tilde{s}(∙)\neq s(∙)$, since the actual explored state has the newly generated in/out-flows at the next time step. Thus, in the training of MD-model, we assign the discount factor *γ* to 0 in order to avoid misestimating the Q-value of the next time step, *Q*(*s*_*t*+1_, *a*_*t*+1_). Note that the estimation of the Q-function in the online learning method uses the bootstrapping as in (7),

$$Q(s_{t},a_{t})=(1-\alpha )Q(s_{t},a_{t})+\alpha \{r_{t+1}+\gamma Q(s_{t+1},a_{t+1})\}$$ (7)

so that an accurately-estimated *Q*(*s*_*t*+1_, ∙) is necessary to estimate *Q*(*s*_*t*_, ∙). In this case, assigning *γ* = 0 enables the model to be free from misestimating Q-value for the unexplored experiences.

## Validation of perimeter control models

The goal of this section is to validate the proposed models by evaluating with the test scenarios at different levels. First, the details of the experiments for the macroscopic traffic simulation, the comparison control methods, and the performance measures are described. Next, the training results for the proposed models are given and finally, the validation results of the proposed models on the different test demand scenarios are shown.

### 1. Experimental setup

#### Setup for macroscopic traffic simulation

To simulate the macroscopic behavior of the transfer flow, we use the agent-based network transmission model (ANTM) developed by [18]. This model can describe the aggregated traffic flow dynamics of the large-scale urban area using the concept of MFDs to represent the network properties. Besides, ANTM enables to apply various perimeter control methods as it considers the effects of travel demand patterns and boundary capacities on the shape changes of MFDs [19], which are directly induced by the perimeter control actions. Moreover, the associated RL model can obtain the speed-up simulation data as the adoption of the cell transmission model (CTM) [41] in the network modeling simplifies the computational complexity.

**Fig 2:**
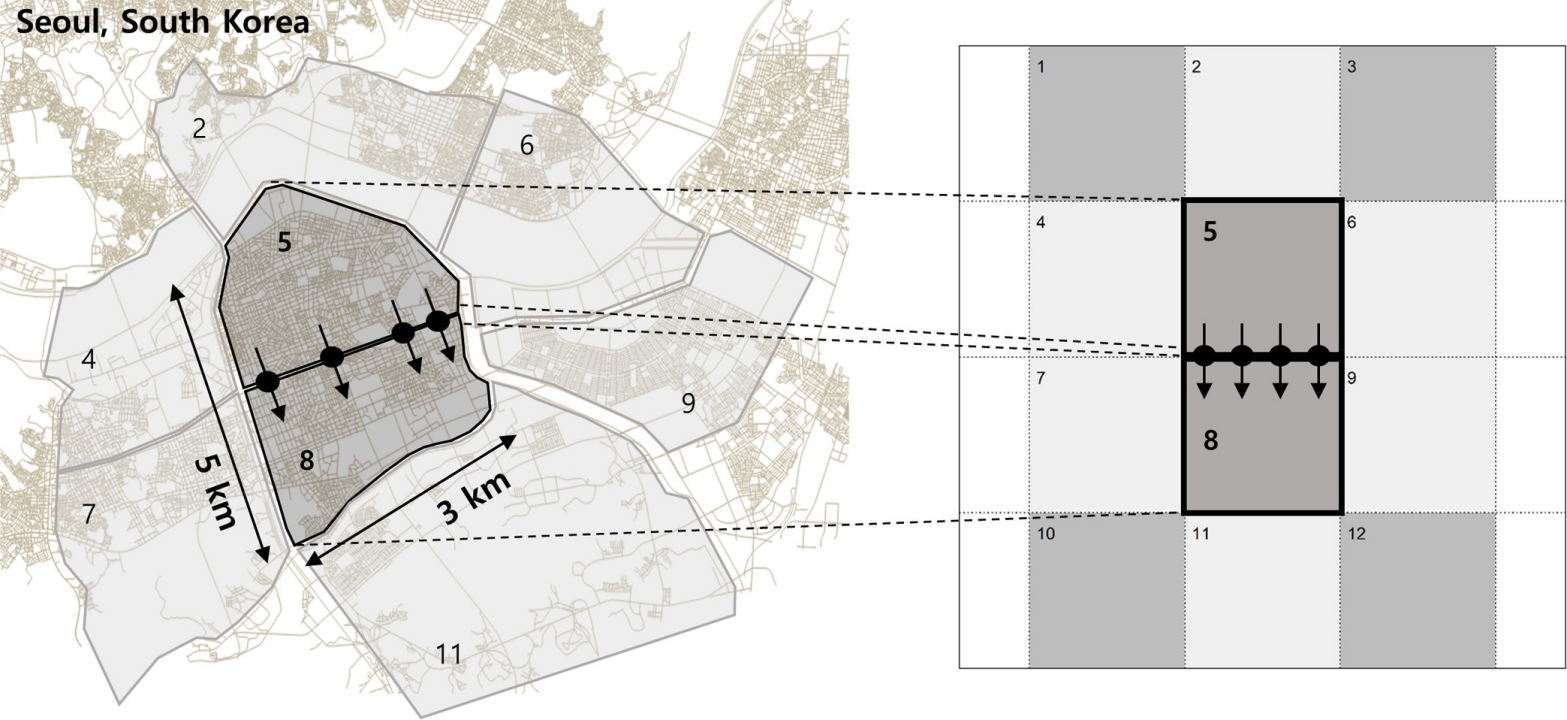
The target area (Gangnam district, Seoul, South Korea). (Drawn by authors using QGIS program, data source: public data portal of South Korea, the data are free source for everyone, the authors did not have any special privileges that others would have in obtaining the data, Data source: https://www.data.go.kr/data/15025526/fileData.do).

Next, we set up the geometry and travel demand scenario for the macroscopic traffic simulation. In this study, the area of Gangnam district in Seoul, South Korea is chosen as the target region, because this area has the road network in an almost grid structure as shown in Fig 2. The networks numbered 5 and 8 are the areas of interest and the rests are exogenous boundary networks. However, we restrict the scope of perimeter control to a single agent problem in order to focus on the validation of model behaviors. Therefore, we do not consider the effect of coordination of multi-agents or the route choice, or the geometric inhomogeneity. The target perimeter controllers are located along the border between the network 5 and 8, which regulate the one-directional transfer flow from the network 5 to 8 (hereinafter referred to as “5→8”). The other controllers are assumed to be fixed (non-controllable). This control method can be considered as the one-sided gating problem. Next, the model is trained with one training demand scenario, which is defined to have a strongly biased directional pattern of 5→8 with constant flow rate. The parameters for the geometry and the training demand scenario are shown in Table 1. The total time length for the training demand scenario is 3 hours and the traffic volume of 120,000veh is distributed with a certain pattern defined in Table 1.

**Table 1:** Hyper-parameters for the macroscopic traffic simulation. (Written by authors).

Geometric Parameters				Demand Scenario Parameters			
Parameters		Value	Unit	Parameters		Value	Unit
*C*_*i*_	*Capacity*	28,800	veh	*Total simulation time*		180	min
c	*Cycle length*	120	sec	*Time step*		2	min
*n*_*l*_	*# lanes*	12		*Total demand*		120,000	veh
*v*_*f*_	*Free speed*	7.2	*hr*^−1^	*Distribution*		uniform	
*π*	*Wave speed*	2.88	*hr*^−1^	*Major demand pattern*		5 → 8	
*n*_*crit*_	*Critical density*	[4,000, 7,000]	veh	*% of major demand pattern*	*Source*	20%	Net 5
*n*_*jam*_	*Jam density*	17,000	veh		*Sink*	80%	Net 8
*q*_*jam*_	*Jam flow*	720	veh/*hr*	*% of local in/outflow rates*	*Inflow*	60%	
*n*_*i*_(0)	*Initial density*	1,000	veh		*Outflow*	1%	

#### Test demand scenarios

In the validation, the performances of the proposed models are evaluated with three test demand scenarios at different levels to investigate how the model adapts to a new demand scenario as in [42,43]. These test scenarios are set to have different patterns of traveling demand from that of the training scenario to generate a “never-before-seen” traffic state. We classify each traveling pattern with two standards: the direction of the major traveling pattern and the traffic loading phase, since the travel demand scenario is defined by the two factors, the O-D pairs and their generation rate (or arrival rate).

**Fig 3:**
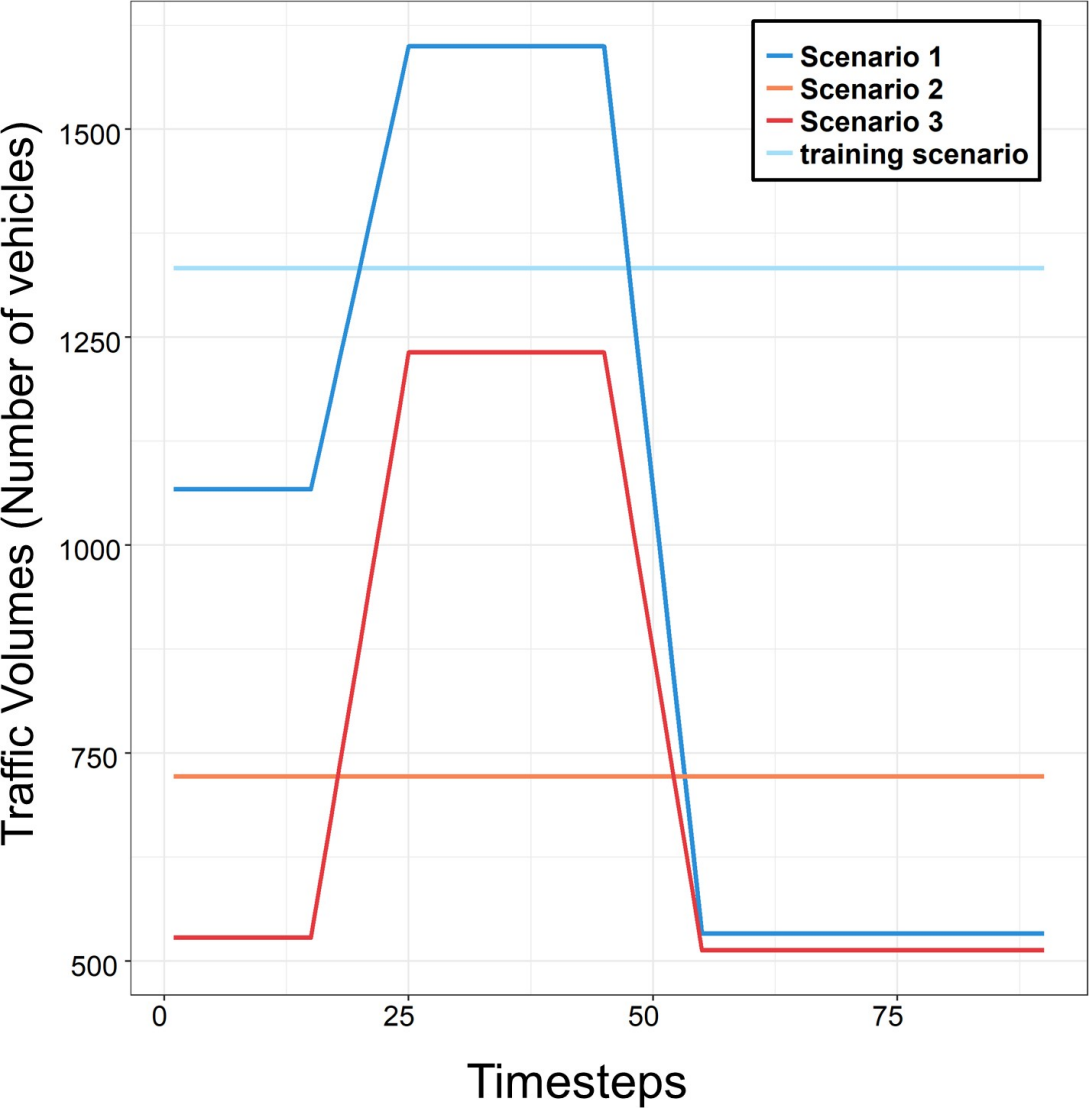
Traffic volumes of the test demand scenarios. (Drawn by authors).

The major traveling pattern (5→8) of the first test scenario is designed to have the same direction with that of the training scenario. However, the first test scenario has non-constant traffic loading/unloading phases that makes the agent explore similar traffic states to those explored in the training session. On the other hand, the second and third test scenarios reverse the direction of the major traveling pattern so that the majority of demand travels 8→5. The desirable action may induce the blockage of the transferring demand of 5→8 when network 8 is congested since the scope of perimeter control is restricted only to the single agent gating problem, which increases the vehicle accumulation in network 5. Therefore, we track the action values in the scenarios, which have a reversed direction of traveling pattern. In addition, one should note that the model may not handle such a large amount of traffic volume in these cases since the defined single agent model only regulates the transferring flow of 5→8. Accordingly, the total traffic volume is lowered. Fig 3 shows the traffic volumes of the test demand scenarios.

#### Comparison control methods and performance measures

The model performance is compared with that of fixed control and the greedy control. Fixed control is the simplest one that fixes the action with the mean value of the action space A. Greedy control is a type of feedback control that dynamically sets the action value as its maximum or minimum according to the traffic state. The action selection rule is shown in (8),

$$a_{i\to j}(t)=\left\{ \begin{array}{c} \max\;A,\;if\;n_{j}(t)\leq n_{j,cr}\\ \min\;A,\;if\;n_{j}(t)>n_{j,cr} \end{array}\right.$$ (8)

where *n*_*j*,*cr*_ represents the critical accumulation (critical density) of the sink network *j*. Note that the given rule in (8) is a modified for the single agent problem. Its complete description can be found in [18]. On the other hand, the action value for the RL control is greedily chosen from its Q-function as in (9),

$$a(t)=\underset{a\prime }{\mathrm{argmax}}\;Q(s_{t},a\prime )$$ (9)

To evaluate how the given control models manage the traffic demands effectively, we use local- and system-level performance measures. For the local-level measures, the vehicle accumulation and the outflow rate of the source network are given. One can note that the outflow rate is proportional to the vehicle accumulation in an uncongested situation, however, this relationship breaks if the vehicle accumulation exceeds the range of critical density. Moreover, the total travel time is considered as a system-level measure.

### 2. Training results

#### Model description

The proposed four types of perimeter control models are trained using deep Q-network (DQN). DQN is a widely used training method applied on a discrete action space. Accordingly, we assume that the action space A has discrete values of green time ratios as A={0.2,0.4,0.6,0.8}, which does not contain 0 or 1 to consider the minimum (or maximum) green time. A deep neural network with parameters *θ* approximates the Q-function of the state-action pair (*s*,*a*), as *Q*(*s*,*a*;*θ*).

**Fig 4:**
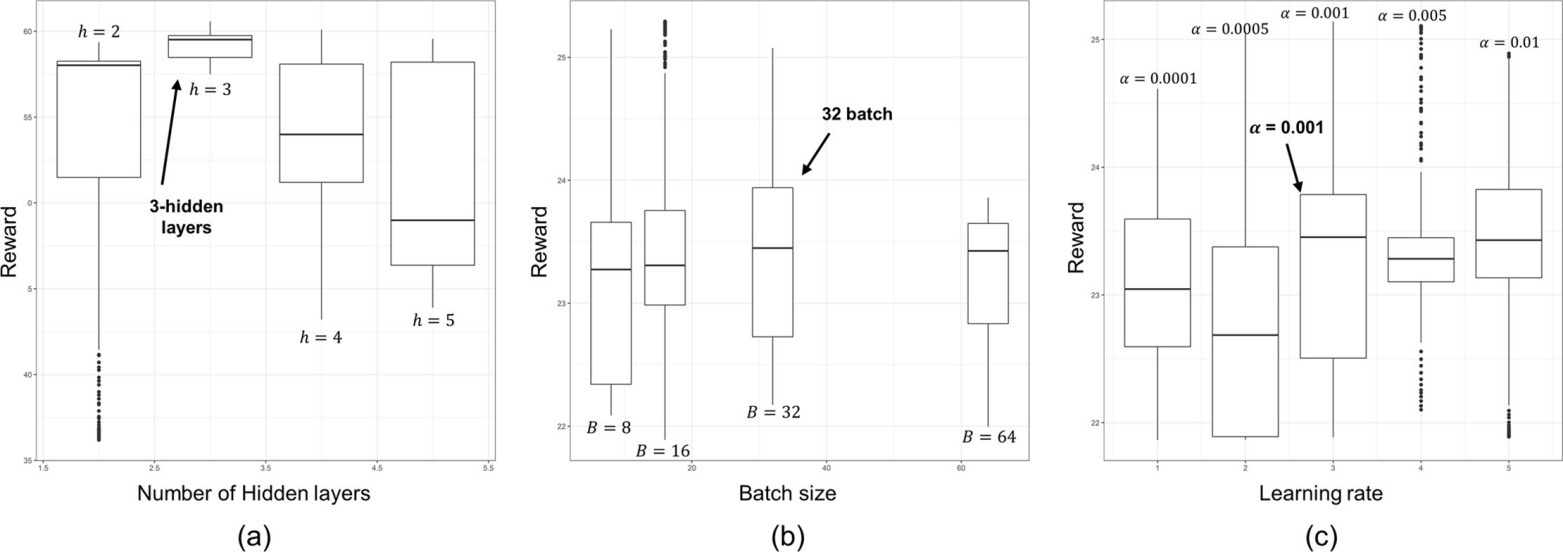
Training performances under different settings of hyper-parameters; (a) number of hidden layers; (b) size of replay buffer; (c) learning rate. (Drawn by authors using R program).

We employ a trial-and-error method [44] to determine the proper hyper-parameters that affect the model efficiencies such as the number of hidden layers, the size of the replay buffer, the learning rate. Fig 4 represents the training performances with respect to the candidate of each hyper-parameter. To show the training performance, the range of the reward curve is plotted. The candidate and proper value of each hyper-parameter are shown in Table 2. The training performance according to the number of hidden layers shows the best result when *h* = 3, where *h* is the number of hidden layers. One can note that the model fails to converge when *h* = 5, as it has a lower mean value and its range is widely spread as seen in Fig 4(A). The candidate for the size of the replay buffer is determined by the group of {2^*n*^} due to the memory. Similarly, the proper value for the size of the replay buffer is chosen as *B* = 32, where *B* is the size of the replay buffer, as the mean value for the range of rewards is larger than the others as shown in Fig 4(B). In the same manner, the proper value for the learning rate *α* is chosen as *α* = 0.001 as seen in Fig 4(C).

**Table 2:** Hyper-parameter settings for the RL model. (Written by authors).

Parameters	Candidate	Chosen value
Number of hidden layers	{2, 3, 4, 5}	3
Size of replay buffer	{8, 16, 32, 64}	32
Learning rate	{0.0001, 0.0005, 0.001, 0.005, 0.01}	0.001

A target network *Q*(*s*,*a*;*θ*^−^) is also used, and this network is updated only at the end of each episode. The loss function is defined together with this target network as in (10)

$$L(t)={\left(r(t)+\gamma \;\underset{a\prime }{\max}\;Q(s_{t+1},a\prime ;\theta^{-})-Q(s_{t},a;\theta )\right)}^{2}$$ (10)

The initial *ϵ* value, which is the probability of the exploration, is set as 1.0 to take random actions entirely, and it gradually decays to 0.01 as the time elapses. The discount factor *γ* in (2) is fixed to 1.0 as the length of each episode is finitely same.

#### Training results for the proposed models

**Fig 5:**
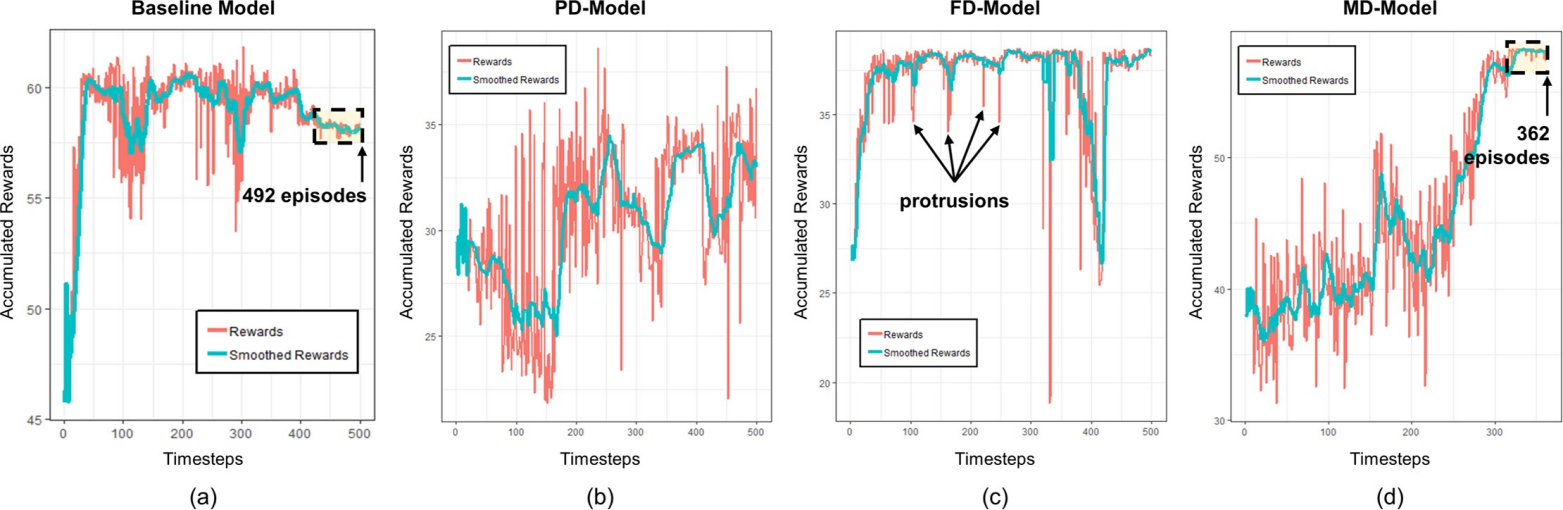
Training results; (a) Baseline model; (b) PD-model; (c) FD-model; (d) MD-model. (Drawn by authors using R program).

To determine the completion of the training, we observe the reward curve of each model. Fig 5 shows the episode accumulated rewards. If the curve converges after some episodes, the corresponding model is considered to be valid. The stopping criteria are set (by heuristic) when the rate of changes of the rewards remains within a certain range for 80 successive episodes before reaching the end of the training. Fig 5(A) shows the training result of the baseline model, while Fig 5(B)~5(D) show the results for PD-, FD- and MD-models, respectively. Within the same stopping criteria, the baseline model completes the training after 492 episodes and MD-model ends after 362 episodes. On the other hand, PD- and FD-models fail to converge. Note that the reward curve for FD-model has several protrusions in which the stopping criteria is not satisfied. Thus, we can conclude that only the baseline model and MD-model are valid within the given training demand scenario.

### 3. Validation results

#### Test scenario 1

**Fig 6:**
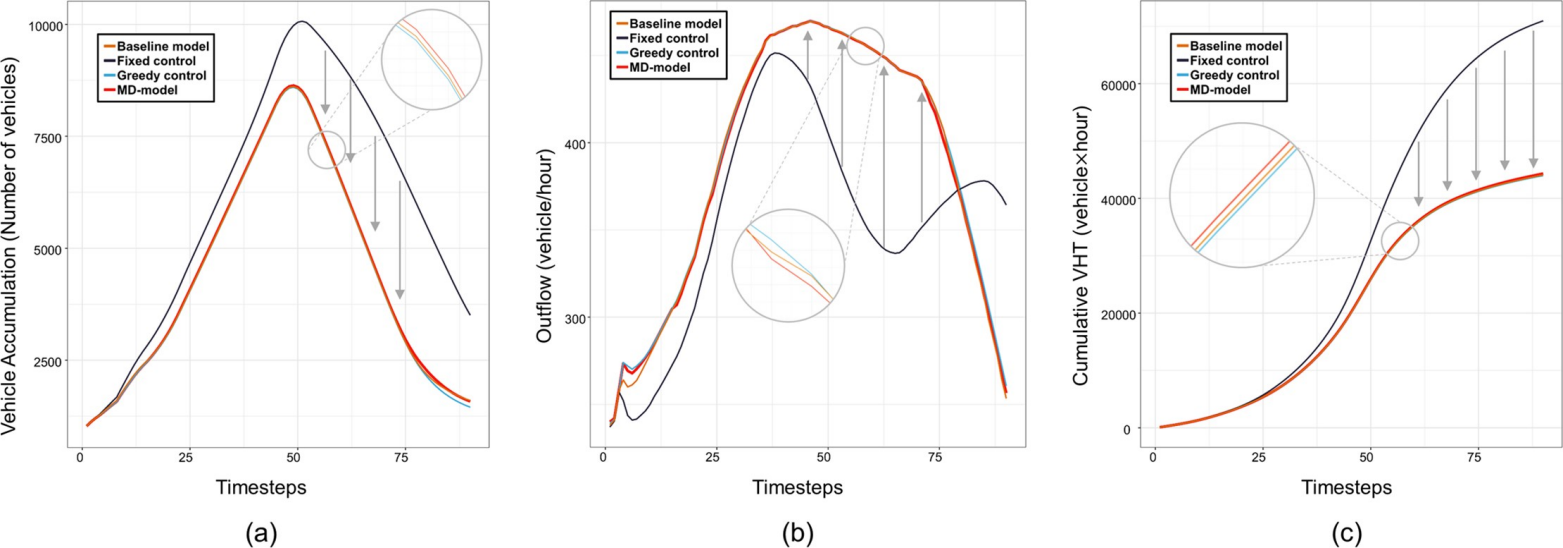
Model validation results of first test scenario; (a) Vehicle accumulation of network 5; (b) Outflow rate of network 5; (c) Cumulative total travel times (VHT). (Drawn by authors using R program).

The validation results for the first test scenario are shown in Fig 6. The first two graphs show the vehicle accumulation and the outflow rate of network 6, respectively, which are local-level performances. On the other hand, the last graph shows the total travel time of the whole networks, which is a system-level performance. Every control method except fixed control maximizes the outflow rate of the transferring demand 5→8 as shown in Fig 6(B) since the target controllers only regulate one-directional transfer flow 5→8 and the majority of demands heads 5→8. Accordingly, network 5 maintains a low vehicle accumulation except for the case of fixed control. The sudden drop of outflow rate for fixed control in Fig 6(B) results from the oversaturation of network 5, which induces a gap in the vehicle accumulation during the unloading phase compared to the other control methods as seen in Fig 6(A). The failure of fixed control also leads to the increased total travel time since it makes network 5 fall into the gridlock. On the other hand, the baseline model, MD-model, and greedy control effectively reduce the total travel time as in Fig 6(C).

#### Test scenario 2, 3

**Fig 7:**
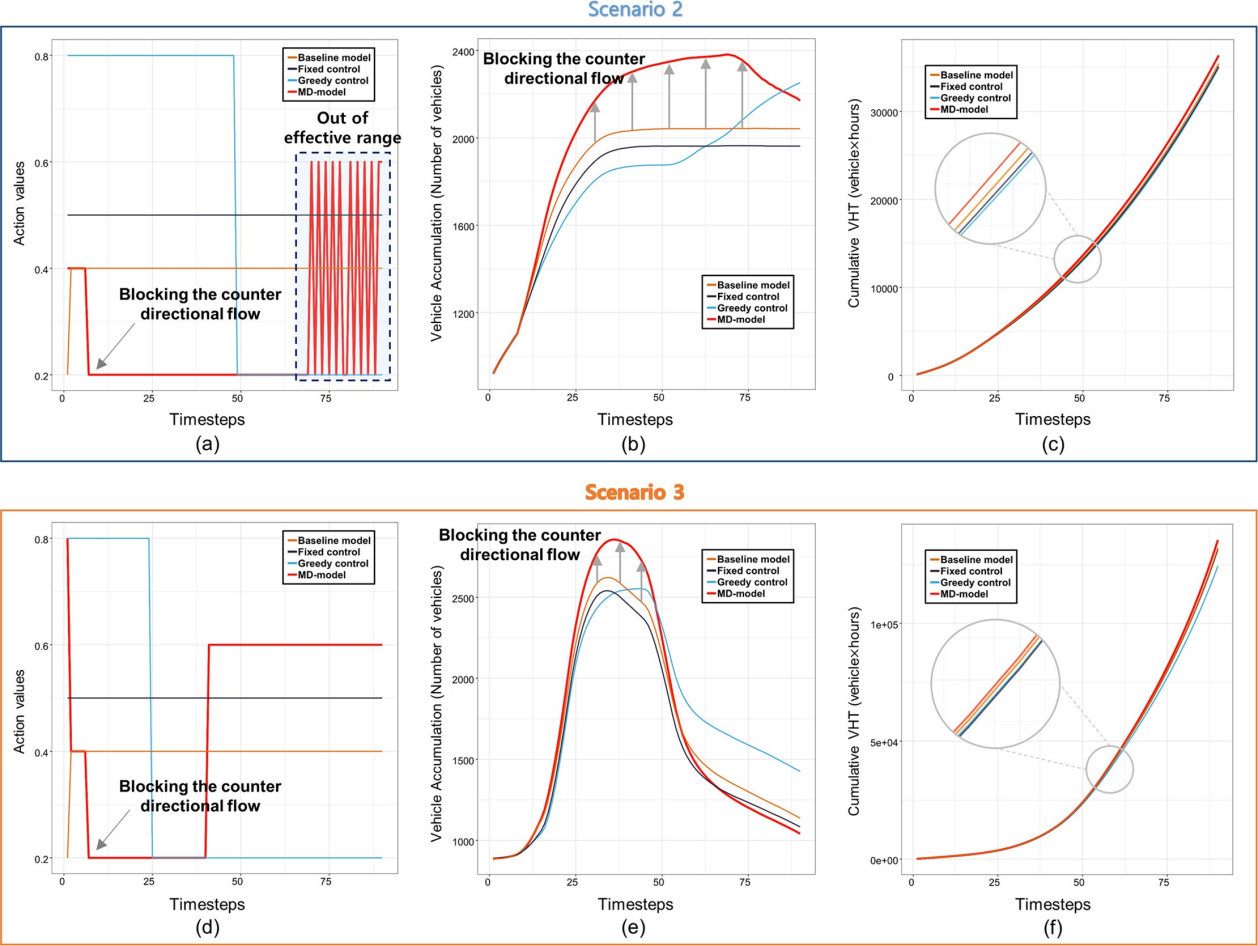
Model validation results of second and third test scenarios; (a) Action values for second test scenario; (b) Vehicle accumulation of network 5 for second test scenario; (c) Cumulative total travel times (VHT) for second test scenario; (d) Action values for third test scenario; (e) Vehicle accumulation of network 5 for third test scenario; (f) Cumulative total travel times (VHT) for third test scenario. (Drawn by authors using R program).

The validation results for the second and third test scenarios are shown in Fig 7. In both test scenarios, the action value for greedy control initially takes the maximum at the early stage. However, the value suddenly drops to the minimum after network 8 reaching its critical level of vehicle accumulation as depicted in Fig 7(A) and 7(D). This implies that greedy control dynamically changes the policy to prevent the oversaturation of the sink network (network 8). On the other hand, the action for fixed control and the baseline model take the constant value throughout the entire time step. This implies that the baseline model cannot adapt to the new demand scenarios.

Meanwhile, MD-model most proactively blocks the travel demand 5→8 in both test scenarios by taking the minimum action value earlier than greedy control as shown in Fig 7(A) and 7(D). However, MD-model fails to adapt to the traffic states explored in the later stage of the second test scenario as there are fluctuations in the action values as seen in Fig 7(A). This can infer that MD-model cannot adapts to new situations much different from the training demand scenario. However, it adapts to the third test scenario effectively since it proactively blocks the travel demand 5→8 during the traffic loading phase and gradually releases during the unloading phase to mitigate the congestion in network 5 as seen in Fig 7(D).

Moreover, the vehicle accumulation of network 5 grows faster in the case of MD-model than others as observed in Fig 7(B) and 7(E), because the travel demand of 5→8 are blocked. However, the differences in total travel time are not remarkable as in Fig 7(C) and 7(F) since the traffic volumes are lowered. As a result, MD-model adapts relatively well to new situations induced by the test scenarios. The explanatory variables in the design of MD-model make the model more effective than the baseline model.

## Conclusion

Perimeter control is an emerging alternative for traffic signal control, which regulates the network-wide traffic demands based on the novel concept of MFDs. Although many researches have been conducted to optimize perimeter control using model-based approaches such as feedback control or MPC, there has been a growing interest in developing an effective model for a large-scale urban area. However, there are several limitations when considering their application to a large-scale urban area because the model-based approaches may not be scalable to multiple regions and inappropriate for handling various effects caused by the shape changes of MFDs. Therefore, we propose a model-free and data-driven approach that combines reinforcement learning (RL) with the macroscopic traffic simulation based on the recently developed network transmission model. This research is the first to introduce RL approach for perimeter control. We experimentally investigate the effective designs of RL model under a well-controlled environment.

We propose four perimeter control models using different designs. The first one is the baseline model and it only contains density-related factors. We additionally append the parameters for the travel demand information to the state as the explanatory variables to construct the other three models referred to PD-, FD-, and MD-models. These models have different macroscopic traffic variables and parametrizations. The results of the training with a fixed travel demand scenario in the macroscopic traffic simulation show that only the baseline model and MD-model are valid. Next, the proposed models are validated with the test demand scenarios at different levels to investigate their ability to adapt to a new situation. Experimental results show that MD-model adapts relatively well to a new situation even if there are some deviations from the effective range of the model in the second test scenario. The baseline model fails to adapt to the test scenarios having reversed travel demand patterns, while MD-model proactively adapts to these scenarios than any other control method. Thus, we can conclude that the parametrization of the travel demand information makes the model more adaptive to a new situation.

However, there are still two remaining issues for constructing a more effective perimeter control based on RL. First, MD-model may not perform as a competent multi-stage optimizer due to the assignment of *γ* = 0 in (5) and (6). In this case, the well-trained policy from the simulated experiences does not consider the expected future rewards and it only uses the current single-shot Q-value. Thus, a new design of traffic simulation and training demand scenario should be considered. Second, a reduced expression for the travel demand patterns should be studied. When the model contains the full demand information of every direction, the dimension of the state space becomes high. Then, the model may require an extreme number of training demand scenarios to explore every dimension of its state space. Hence, there is a need to acquire principal factors for travel demand patterns to reduce the dimension of the searching space.

## References

[1] Sims A. the Sydney Co-ordinated Adaptive Traffic (Scat) System. Publ Aust Road Res Board. 1979;29: 130–137. Available: http://trid.trb.org/view.aspx?id=147832

[2] Diakaki C, Papageorgiou M, Aboudolas K. A multivariable regulator approach to traffic-responsive network- Wide signal control. Control Eng Pract. 2002;10: 183–195. 10.1016/S0967-0661(01)00121-6

[3] Chang J, Bertoli B, Xin W. New signal control optimization policy for oversaturated arterial systems. Transportation Research Board. 2010 pp. 10–3760.

[4] Daganzo CF. Urban gridlock: Macroscopic modeling and mitigation approaches. Transp Res Part B Methodol. 2007;41: 49–62. 10.1016/j.trb.2006.03.001

[5] Geroliminis N. Macroscopic modeling of traffic in cities. Transportation Research Board. 2007 pp. 07–0413.

[6] Geroliminis N, Daganzo CF. Existence of urban-scale macroscopic fundamental diagrams: Some experimental findings. Transp Res Part B Methodol. 2008;42: 759–770. 10.1016/j.trb.2008.02.002

[7] Keyvan-Ekbatani M, Kouvelas A, Papamichail I, Papageorgiou M. Exploiting the fundamental diagram of urban networks for feedback-based gating. Transp Res Part B Methodol. 2012;46: 1393–1403. 10.1016/j.trb.2012.06.008

[8] Csikos A, Tettamanti T, Varga I. Nonlinear gating control for urban road traffic network using the network fundamental diagram. J Adv Transp. 2015;49: 597–615. 10.1002/atr

[9] Haddad J, Shraiber A. Robust perimeter control design for an urban region. Transp Res Part B Methodol. 2014;68: 315–332. 10.1016/j.trb.2014.06.010

[10] Haddad J, Geroliminis N. On the stability of traffic perimeter control in two-region urban cities. Transp Res Part B Methodol. 2012;46: 1159–1176. 10.1016/j.trb.2012.04.004

[11] Haddad J. Robust Constrained Control of Uncertain Macroscopic Fundamental Diagram Networks. Transp Res Procedia. 2015;7: 669–688. 10.1016/j.trpro.2015.06.035

[12] Aboudolas K, Geroliminis N. Perimeter and boundary flow control in multi-reservoir heterogeneous networks. Transp Res Part B Methodol. 2013;55: 265–281. 10.1016/j.trb.2013.07.003

[13] Kouvelas A, Saeedmanesh M, Geroliminis N. Enhancing model-based feedback perimeter control with data-driven online adaptive optimization. Transp Res Part B Methodol. 2017;96: 26–45. 10.1016/j.trb.2016.10.011

[14] Hajiahmadi M, Haddad J, De Schutter B, Geroliminis N. Optimal hybrid perimeter and switching plans control for urban traffic networks. IEEE Trans Control Syst Technol. 2014;23: 464–478. 10.23919/ecc.2013.6669572

[15] Ramezani M, Haddad J, Geroliminis N. Dynamics of heterogeneity in urban networks: Aggregated traffic modeling and hierarchical control. Transp Res Part B Methodol. 2015;74: 1–19. 10.1016/j.trb.2014.12.010

[16] Yang K, Zheng N, Menendez M. Multi-scale perimeter control approach in a connected-vehicle environment. Transp Res Part C Emerg Technol. 2017;94: 32–49. 10.1016/j.trc.2017.08.014 PMC6134872 30220823

[17] Ernst D, Glavic M, Capitanescu F, Wehenkel L. Reinforcement learning versus model predictive control: A comparison on a power system problem. IEEE Trans Syst Man, Cybern Part B Cybern. 2008;39: 517–529. 10.1109/TSMCB.2008.2007630 19095542

[18] Kim S, Tak S, Yeo H. Agent-based network transmission model using the properties of macroscopic fundamental diagram. Transp Res Part C Emerg Technol. 2018;93: 79–101. 10.1016/j.trc.2018.05.024

[19] Kim S, Tak S, Yeo H. Investigating transfer flow between urban networks based on a macroscopic fundamental diagram. Transp Res Rec. 2018;2672: 75–85. 10.1177/0361198118778927

[20] Buisson C, Ladier C. Exploring the impact of homogeneity of traffic measurements on the existence of macroscopic fundamental diagrams. Transp Res Rec. 2009;2124: 127–136. 10.3141/2124-12

[21] Geroliminis N, Sun J. Properties of a well-defined macroscopic fundamental diagram for urban traffic. Transp Res Part B Methodol. 2011;45: 605–617. 10.1016/j.trb.2010.11.004

[22] Mazloumian A, Geroliminis N, Helbing D. The spatial variability of vehicle densities as determinant of urban network capacity. Philos Trans R Soc A Math Phys Eng Sci. 2010;368: 4627–4647. 10.1098/rsta.2010.0099 20819825

[23] Ji Y, Daamen W, Hoogendoorn S, Hoogendoorn-Lanser S, Qian X. Investigating the shape of the macroscopic fundamental diagram using simulation data. Transp Res Rec. 2010;2161: 40–48. 10.3141/2161-05

[24] Knoop VL, Van Lint H, Hoogendoorn SP. Traffic dynamics: Its impact on the Macroscopic Fundamental Diagram. Phys A Stat Mech its Appl. 2015;438: 236–250. 10.1016/j.physa.2015.06.016

[25] Geroliminis N, Sun J. Hysteresis phenomena of a macroscopic fundamental diagram in freeway networks. Procedia—Social and Behavioral Sciences. 2011 pp. 213–228. 10.1016/j.sbspro.2011.04.515

[26] Zhang L, Garoni TM, de Gier J. A comparative study of Macroscopic Fundamental Diagrams of arterial road networks governed by adaptive traffic signal systems. Transp Res Part B Methodol. 2013;49: 1–23. 10.1016/j.trb.2012.12.002

[27] Geroliminis N, Haddad J, Ramezani M. Optimal perimeter control for two urban regions with macroscopic fundamental diagrams: A model predictive approach. IEEE Trans Intell Transp Syst. 2013;14: 348–359. 10.1109/TITS.2012.2216877

[28] El-Tantawy S, Abdulhai B. Multi-agent reinforcement learning for integrated network of adaptive traffic signal controllers (MARLIN-ATSC). IEEE Trans Intell Transp Syst. 2013;14: 1140–1150. 10.1109/ITSC.2012.6338707

[29] Casas N. Deep Deterministic Policy Gradient for Urban Traffic Light Control. arXiv Prepr. 2017;1703 09035: 1–12. Available: http://arxiv.org/abs/1703.09035

[30] Chu T, Wang J, Codeca L, Li Z. Multi-Agent Deep Reinforcement Learning for Large-Scale Traffic Signal Control. IEEE Trans Intell Transp Syst. 2019 10.1109/TITS.2018.2868182

[31] Knoop VL, Hoogendoorn SP. An area-aggregated dynamic traffic simulation model. Eur J Transp Infrastruct Res. 2015;15: 226–242. 10.18757/ejtir.2015.15.2.3070

[32] Ma D, Sheng B, Wang D, Jin S, Song X. Prompt prediction of spillovers in urban street networks by using rolling time occupancy data. Transp A Transp Sci. 2019;15: 1041–1060. 10.1080/23249935.2018.1563920

[33] Aboudolas K, Geroliminis N. Feedback perimeter control for multi-region large-scale congested networks. European Control Conference (ECC). 2013. pp. 3506–3511.

[34] Haddad J, Mirkin B. Coordinated distributed adaptive perimeter control for large-scale urban road networks. Transp Res Part C Emerg Technol. 2017;77: 495–515. 10.1016/j.trc.2016.12.002

[35] Keyvan-Ekbatani M, Yildirimoglu M, Geroliminis N, Papageorgiou M. Traffic signal perimeter control with multiple boundaries for large urban networks. IEEE Conference on Intelligent Transportation Systems, Proceedings, ITSC. 2013. pp. 1004–1009. 10.1109/ITSC.2013.6728363

[36] Kouvelas A, Saeedmanesh M, Geroliminis N. Feedback Perimeter Control for Heterogeneous Urban Networks Using Adaptive Optimization. IEEE Conference on Intelligent Transportation Systems, Proceedings, ITSC. 2015. pp. 882–887. 10.1109/ITSC.2015.148

[37] Yang K, Zheng N, Menendez M. Multi-scale Perimeter Control Approach in a Connected-Vehicle Environment. Transp Res Procedia. 2017;23: 101–120. 10.1016/j.trpro.2017.05.007

[38] Abdulhai B, Pringle R, Karakoulas GJ. Reinforcement learning for true adaptive traffic signal control. J Transp Eng. 2003;129: 278–285. 10.1061/(ASCE)0733-947X(2003)129:3(278)

[39] Gao J, Shen Y, Liu J, Ito M, Shiratori N. Adaptive Traffic Signal Control: Deep Reinforcement Learning Algorithm with Experience Replay and Target Network. arXiv Prepr. 2017;1705 02755 Available: http://arxiv.org/abs/1705.02755

[40] Mnih V, Kavukcuoglu K, Silver D, Rusu AA, Veness J, Bellemare MG, et al Human-level control through deep reinforcement learning. Nature. 2015;518: 529–533. 10.1038/nature14236 25719670

[41] Daganzo CF. The cell transmission model: A dynamic representation of highway traffic consistent with the hydrodynamic theory. Transp Res Part B. 1993;28: 269–287. 10.1016/0191-2615(94)90002-7

[42] Chen X, Wang S, Shi C, Wu H, Zhao J, Fu J. Robust ship tracking via multi-view learning and sparse representation. J Navig. 2019;72: 176–192. 10.1017/S0373463318000504

[43] Chen X, Xu X, Yang Y, Wu H, Tang J, Zhao J. Augmented Ship Tracking under Occlusion Conditions from Maritime Surveillance Videos. IEEE Access. 2020;8: 42884–42897. 10.1109/ACCESS.2020.2978054

[44] Chen X, Yang Y, Wang S, Wu H, Tang J, Zhao J, et al Ship Type Recognition via a Coarse-to-Fine Cascaded Convolution Neural Network. J Navig. 2020; 1–20. 10.1017/s0373463319000900

